# Hacking the Permeability Barrier of Gram-Negative Bacteria

**DOI:** 10.1021/acscentsci.2c00750

**Published:** 2022-08-10

**Authors:** Ziyang Zhang

**Affiliations:** Department of Chemistry, University of California, Berkeley, California 94720, United States

Antibiotics save numerous lives. Although bacteria inevitably develop resistance to existing antibiotics thanks to natural evolution, humanity has been largely successful in keeping up with these evolving pathogens with the continuous discovery of new antibiotics—until recently. With the rapid rise of multidrug-resistant Gram-negative bacteria and a diminishing antibiotic pipeline, many fear that we are losing the tug-of-war between bacterial resistance and human innovation. Gram-negative bacteria account for the lion’s share of drug-resistant pathogens that cause life-threatening infections—4 of the 6 ESKAPE pathogens^1^ and 9 of the 12 priority pathogens designated by WHO^2^ are Gram-negative bacteria. However, finding new antibiotics against Gram-negative bacteria is no easy feat: since 1968, no new major classes of antibiotics have been approved by the FDA to treat deadly Gram-negative infections.

Writing in this issue of *ACS Cent. Sci.*, Parker, Cain et al. report the discovery of fabimycin, a new antibiotic candidate against Gram-negative pathogens that acts by inhibiting a critical enzyme in bacterial fatty acid biosynthesis—FabI.^3^ Using a set of physiochemical guidelines to improve compound accumulation in Gram-negative bacteria and structure-guided chemical synthesis, the researchers successfully converted a previously reported FabI inhibitor with no Gram-negative activity into one that kills a wide array of Gram-negative clinical isolates.

**Figure 1:**
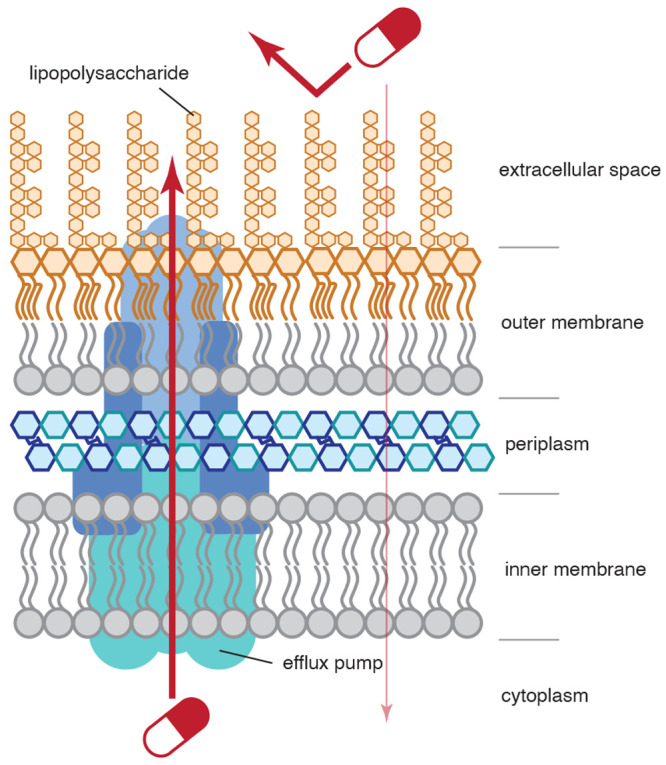
Illustration of the Gram-negative cell wall and the barriers that prevent the entry and retention of antibiotics.

Gram-negative bacteria are born with advantages to resist antibiotic action (Figure 1). At least two distinct features of the Gram-negative cell wall helps it repel small-molecule antibiotics much more efficiently than its Gram-positive counterpart.^4^ First, the outer membrane, an asymmetric lipid bilayer tightly coated by lipopolysaccharides, serves as a powerful barrier that blocks compound *entry*. Second, even if the antibiotic compound makes its way inside the outer membrane, multidrug efflux pumps can remove antibiotics from both the periplasm and the cytoplasm to prevent their *retention*. Acting synergistically, the outer membrane and efflux pumps can render Gram-negative bacteria impermeable to a wide range of antibiotics.^5^ Fortunately, these barriers are not perfect: some small polar compounds can enter Gram-negative bacteria through small hydrophilic protein tunnels across the outer membrane called porins,^6^ and certain antibiotics can evade the action of efflux pumps.^7^

Our increased understanding of the molecular basis of these entry and retention barriers has fueled an interest to improve the potency of existing antibiotics against Gram-negative bacteria by either (1) compromising or (2) circumventing such barriers. In the first category, efflux pump inhibitors^8^ or outer membrane disrupting agents^9^ have been shown to greatly enhance the potency of multiple antibiotics. In the second category, numerous studies have attempted to define the chemical determinants for antibiotic accumulation in Gram-negative bacteria.^10^ Among these is a study from the researchers’ group in 2017 where Richter et al. directly quantified the intracellular concentrations of more than 100 antibiotics in *Escherichia coli* and performed cheminformatic analysis using 297 calculated molecular descriptors.^11^ This led to the establishment of a set of guidelines that predict compound accumulation in Gram-negative bacteria termed “eNTRy rules”: presence of an ionizable Nitrogen, low Three-dimensionality, and fewer than five Rotatable bonds.

Now, in a case study, Parker, Cain et al. show that these guidelines, when used in concert with a modular chemical synthesis strategy, can support the rapid discovery of potent Gram-negative antibiotic candidates starting from a “Gram-positive only” compound. The key rationale is that the molecular targets of many “Gram-positive only” antibiotics are also present and essential for Gram-negative bacteria. If these antibiotics could attain sufficient intracellular concentration, they would be effective drugs against Gram-negative pathogens. For example, the FabI inhibitor Debio-1452 was inactive in all Gram-negative strains tested, and it showed potent activity in an efflux-deficient *E. coli* strain (Δ*tolC*) with a minimum inhibitory concentration (MIC) of 0.062 mg/mL.

**Figure 2:**
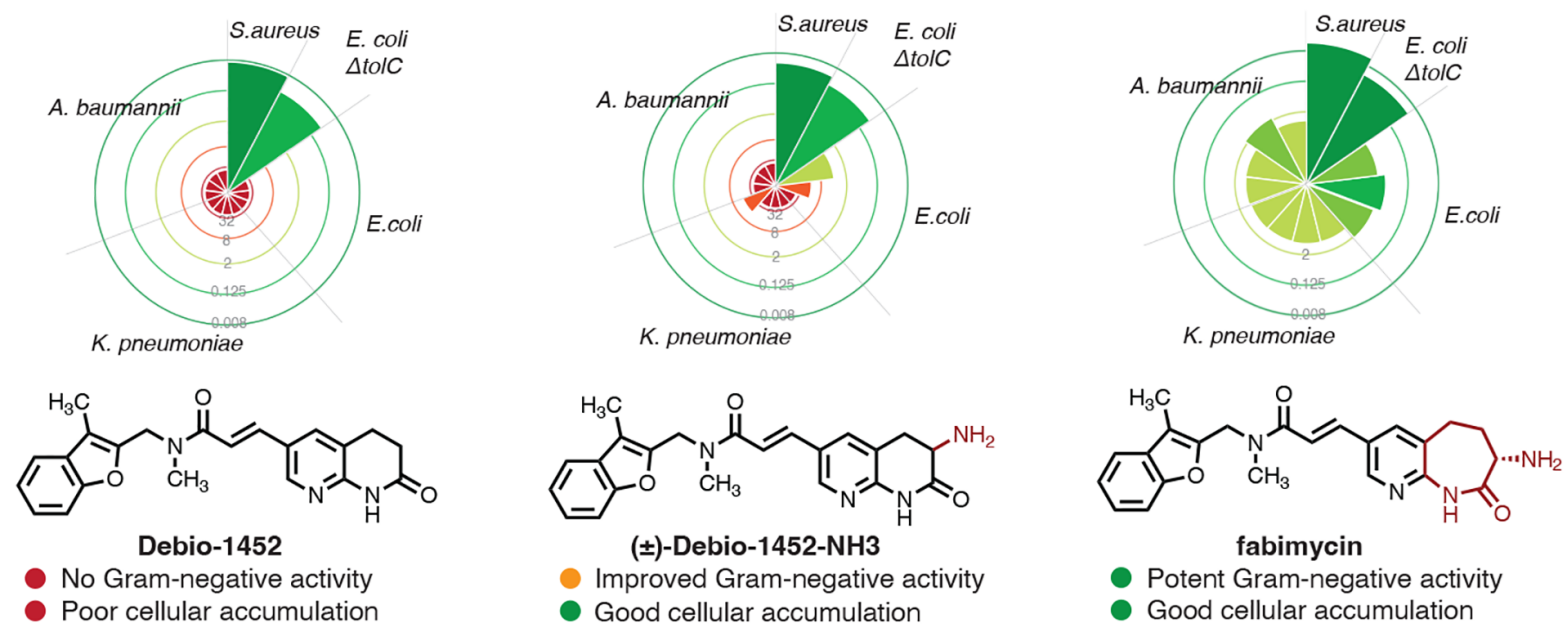
Improving the Gram-negative activity of FabI inhibitors with chemical synthesis and chemoinformatic guidelines. MIC values (μg/mL) in 13 tested bacterial strains are plotted in radar pie charts (Data source: ref (3), Figure 2. Copyright 2022 The Authors. Published by American Chemical Society).

To improve the Gram-negative activity of Debio-1452, the researchers followed the “eNTRy” guidelines and sought to introduce a primary amine to its solvent exposed portion (Figure 2). The researchers previously reported that the addition of an amino group to 6-position of the tetrahydro-1,8-naphthyridine ring led to a moderate improvement of its activity in Gram-negative reference strains. This led Parker, Cain et al. to pursue a modular chemical synthesis strategy by joining two individually variable moieties with a convergent Heck coupling reaction. Their strategy allowed the rapid exploration of molecular diversity and the subsequent identification of fabimycin, a ring-expanded analogue of Debio-1452, which gained potent activity against >200 clinical isolates of Gram-negative strains including *E. coli., Klebsiella pneumoniae*, and *Acinetobacter baumannii*. Fabimycin effectively reduced bacterial load in mice challenged with pneumonia, thigh infection, and urinary tract infections (UTI).

FabI is a critical component of the bacterial fatty acid synthesis pathway (FAS-II), one that is sufficiently different from its mammalian counterpart (FAS-I). This grants FabI inhibitors an inherent therapeutic index as an antibiotic target. Indeed, two FabI inhibitors in clinical use, triclosan and isoniazid, are known to be safe and effective antibiotics. Nevertheless, not all bacteria species are dependent on FabI—for example, *Pseudomonas aeruginosa* express the FabV isoform and therefore are not affected by FabI inhibitors. The narrow spectrum of fabimycin is not necessarily a deal breaker; rather, it could help avoid the elimination of the gut microbiome in patients receiving antibiotic treatment, a common side-effect of broad-spectrum antibiotics that greatly increases the risk of *Clostridioides difficile* infections and inflammatory bowel diseases. Promisingly, fabimycin did not show antibacterial activity among the 38 commensal bacterial strains tested.

A formidable challenge for all new antibiotic candidates is the inevitable development of resistance. Parker, Cain et al. found that although the resistance for fabimycin does develop spontaneously, most of the mutant strains can still be inhibited by a higher, but clinically achievable, concentration of fabimycin. Interestingly, neither the G93V mutation conferring *E. coli* resistance to triclosan, which binds at the same site of FabI as fabimycin, nor the M99T mutation (Q99 in *E. coli*) found with Debio-1542 resistant *Staphylococcus aureus* was detected from the fabimycin selection. Although many additional studies will likely be necessary to assess the full therapeutic potential and susceptibility to clinical resistance of fabimycin, the current data make it an exciting lead for further development of antibiotics against Gram-negative bacteria.

While the “eNTRy” guidelines were instrumental in the genesis of fabimycin, several questions remain whose answers could lead us to an even better understanding of rules governing antibiotic permeability in Gram-negative bacteria. (1) The guidelines do not inform the exact molecular mechanisms of increased accumulation. Does adding an amine to Debio-1452 help with the entry across the outer membrane, or does it make the compound less susceptible to efflux pumps? (2) The guidelines were established from compound accumulation data in *E. coli*. Do different Gram-negative species have their own sets of preferred physiochemical properties given their different outer membrane composition and efflux pump expression? (3) Is the predictive power of the guidelines limited by the currently used chemical descriptors? Would new molecular representations bring additional insights?

One hundred and 13 years ago, on his way to discovering one of the earliest antibiotics in history, salvarsan, Paul Ehrlich opined: “there must be planned chemical synthesis: proceeding from a chemical substance with recognizable activity, making derivatives from it, and then trying each to discover the degree of its activity and effectiveness”.^12^ It is refreshing to see that the same principles remain the cornerstone of antibiotics discovery today. With an expanded toolbox of synthetic methodologies, a refined understanding of bacterial biology, and an unprecedented power of chemoinformatic analysis, we are now ready to accelerate our pace to replenish our antibiotic pipeline. The study by Parker, Cain et al. offers a compelling demonstration of the synergy between chemical synthesis and chemoinformatics in countering the ever-evolving threat of antibacterial resistance.
